# Human stem cell derived beta-like cells engineered to present PD-L1 improve transplant survival in NOD mice carrying human HLA class I

**DOI:** 10.3389/fendo.2022.989815

**Published:** 2022-11-25

**Authors:** Jorge Santini-González, Roberto Castro-Gutierrez, Matthew W. Becker, Chad Rancourt, Holger A. Russ, Edward A. Phelps

**Affiliations:** ^1^ J. Crayton Pruitt Family Department of Biomedical Engineering, University of Florida, Gainesville, FL, United States; ^2^ Barbara Davis Center for Diabetes, Department of Pediatrics, University of Colorado Anschutz Medical Campus, Aurora, CO, United States; ^3^ Animal Care Services, University of Florida, Gainesville, FL, United States

**Keywords:** PD-L1, HLA Class I, type 1 diabetes, stem cell-derived beta cell, tolerance, beta cell replacement, humanized mouse

## Abstract

There is a critical need for therapeutic approaches that combine renewable sources of replacement beta cells with localized immunomodulation to counter recurrence of autoimmunity in type 1 diabetes (T1D). However, there are few examples of animal models to study such approaches that incorporate spontaneous autoimmunity directed against human beta cells rather than allogenic rejection. Here, we address this critical limitation by demonstrating rejection and survival of transplanted human stem cell-derived beta-like cells clusters (sBCs) in a fully immune competent mouse model with matching human HLA class I and spontaneous diabetes development. We engineered localized immune tolerance toward transplanted sBCs *via* inducible cell surface overexpression of PD-L1 (iP-sBCs) with and without deletion of all HLA class I surface molecules *via* beta-2 microglobulin knockout (iP-BKO sBCs). NOD.HLA-A2.1 mice, which lack classical murine MHC I and instead express human HLA-A*02:01, underwent transplantation of 1,000 human HLA-A*02:01 sBCs under the kidney capsule and were separated into HLA-A2 positive iP-sBC and HLA-class I negative iP-BKO sBC groups, each with +/- doxycycline (DOX) induced PD-L1 expression. IVIS imaging showed significantly improved graft survival in mice transplanted with PD-L1 expressing iP-sBC at day 3 post transplantation compared to controls. However, luciferase signal dropped below *in vivo* detection limits by day 14 for all groups in this aggressive immune competent diabetes model. Nonetheless, histological examination revealed significant numbers of surviving insulin^+^/PD-L1^+^ sBCs cells for DOX-treated mice at day 16 post-transplant despite extensive infiltration with high numbers of CD3^+^ and CD45^+^ immune cells. These results show that T cells rapidly infiltrate and attack sBC grafts in this model but that significant numbers of PD-L1 expressing sBCs manage to survive in this harsh immunological environment. This investigation represents one of the first *in vivo* studies recapitulating key aspects of human autoimmune diabetes to test immune tolerance approaches with renewable sources of beta cells.

## Introduction

Type 1 diabetes (T1D) is characterized by the selective destruction of the insulin producing beta cells by self-reactive T-cells (1, 2). Cadaveric islet transplantation has demonstrated the feasibility of beta cell replacement to restore insulin independence in some patients (3, 4). Donor organ scarcity and variable islet quality, recurrence of autoimmunity, and the need for systemic immunosuppression remain obstacles to widespread implementation of islet replacement therapy. To overcome these limitations, we and others have demonstrated the successful generation of stem cell derived beta cells (sBC) from human pluripotent stem cell sources (hPSC), effectively generating an abundant and renewable source of functional human beta cells (5–7). sBCs can control normal blood sugar levels in immune compromised preclinical diabetic animal models highlighting their promise for beta cell replacement therapy (5, 8). This technology has rapidly progressed to human patients. Ongoing clinical trials using stem cell derived pancreatic cells in conjunction with immune barrier technologies have demonstrated promising results (9, 10). In addition, a trial exploring the use of naked sBC pseudo islets with systemic immune suppression recently announced that the first patient to receive the therapy achieved insulin independence at day 270 with half the target dose (11).

To avoid the negative consequences of systemic immunosuppression, islet graft immune barrier materials have been developed such as poly-ethylene glycol (PEG), alginate or permeability-selective membranes (12–17). Although these materials prevent direct interaction between islets and immune cells, the encapsulation material can trigger a foreign body response. In addition, by preventing islet revascularization, encapsulation limits islet function and reduces oxygen and nutrient transport, eventually leading to apoptosis and graft failure (15, 18). Thus, there exists a critical unmet need to engineer local tolerance approaches for renewable sources of beta cells that will facilitate islet engraftment while simultaneously preventing immune destruction of the graft.

Others have provided the proof-of-concept that presentation of programmed death ligand 1 (PD-L1) or Fas ligand on the surface of islets or PEG microgels co-transplanted with islets is sufficient to prevent islet rejection in the allo-transplant (19–22) and xeno-transplant setting (23). However, these studies focused on non-humanized animals, chemically induced (not autoimmune) diabetes, and allo/xenograft mechanisms of rejection. Although, the field has vastly advanced by their contribution, it remains to be determined whether such a strategy could be effective in the setting of spontaneous and sustained islet autoimmunity. Thus, further evaluation of the immune component in the context of a human-like spontaneous autoimmunity and using renewable human beta cells is important.

We set out to investigate a strain of NOD mouse that expresses human HLA-A2 molecules instead of mouse MHC class I for transplantation studies of HLA-A2 matched human sBCs (24). The mouse strain NOD-cMHCI^-/–^A2 is a humanized model that develops spontaneous autoimmune diabetes. The mouse MHC class I genes H2-D1 and H2-K1 are genetically knocked out, and the human HLA Class I transgene HLA-A*02:01 (HLA-A2) is incorporated. Previous work by others identified the HLA-A2.1–restricted autoantigenic epitopes in NOD-A2 mice to be derived from the pancreatic β-cell proteins insulin and IGRP (25–27), which are also targeted by CD8^+^ T cells in human T1D patients expressing this HLA class I variant (28–33). Additionally, T cells from NOD-cMHCI^-/–^A2 mice will target and kill human beta cells from A2 donors in an antigen-specific TCR-HLA matched manner (25). Thus, the autoimmune response in these mice recapitulates aspects of T1D in humans, particularly related to beta cell killing.

Here, we transplanted different experimental groups of HLA-A2 expressing sBC clusters (sBCs) into NOD-cMHCI^-/–^A2 mice and performed longitudinal and end point analysis. sBC clusters were engineered to harbor DOX-inducible PD-L1 expression with and without global HLA class I expression. We observed that DOX-inducible PD-L1 improved the survival of sBC grafts in NOD-cMHCI^-/–^A2 recipients for both HLA class I expressing and HLA class I KO cells. As such, our study provides a critical first step into evaluating the effects of sBC immune modulatory interventions by bioengineering technology in a human HLA-A2 matched animal with spontaneous autoimmune rejection.

## Materials and methods

### hPSC culture and differentiation of stem cell derived beta-like cells

sBCs were generated using methods consistent with our previous publications (34). Undifferentiated hPSC Mel1^INS-GFP^ reporter cells (35) were maintained on hES qualified Matrigel (Corning #354277) in mTeSR+ media (STEMCELL Technologies #05826). Differentiation to stem cell derived beta like cells (sBC) was carried out in suspension‐based, bioreactor magnetic stirring system (Reprocell #ABBWVS03A-6, #ABBWVDW-1013, #ABBWBP03N0S-6) as follows. Confluent hPSC cultures were dissociated into single‐cell suspension by incubation with TrypLE (Gibco #12-604-021) for 8 min at 37 C. Detached cells were quenched with mTESR+ media. Cells were then counted using a MoxiGo II cell counter (Orflow), followed by seeding 0.5 × 10^6^ cells/ml in mTeSR+ media supplemented with 10 μM ROCK inhibitor (Y-27632, R&D Systems #1254-50). Bioreactors were placed on a magnetic stirring system set at 60 rpm in a cell culture incubator with 5% CO_2_ to induce sphere formation for 48 hr. To induce definitive endoderm differentiation, spheres were collected in a 50 ml Falcon tube, allowed to settle by gravity, washed once with RPMI (Gibco #11-875-093) + 0.2% FBS, and re‐suspended in d1 media [RPMI containing 0.2% FBS, 1:5,000 ITS (Gibco #41400-045), 100ng/ml Activin A (R&D Systems #338-AC-01M), and 3 μM CHIR99021 (STEMCELL Technologies #72054)]. Differentiation media was changed daily by letting spheres settle by gravity for 3-10 min. Most supernatant was removed by aspiration; fresh media was added, and bioreactors were placed back on stirrer system. sBC differentiation was based on published protocol (5) with modifications as outlined below. Differentiation medias are as: day 2-3: RPMI containing 0.2% FBS, 1:2,000 ITS, and 100 ng/ml Activin A; d4-5: RPMI containing 2% FBS, 1:1,000 ITS, and 50 ng/ml KGF (Prepotech #100-19-1MG); d6: DMEM with 4.5g/L D-glucose (Gibco #11960-044) containing 1:100 SM1 (STEMCELL Technologies #5711), 1:100 NEAA (Gibco #11140-050), 1mM Sodium Pyruvate (Gibco #11360-070), 1:100 GlutaMAX (Gibco #35050-061), 3 nM TTNPB, (R&D Systems #0761), 250 nM Sant-1 (R&D Systems #1974), 250 nM LDN (STEMCELL Technologies #72149), 30 nM PMA (Sigma Aldrich #P1585-1MG), 50 μg/ml 2-phospho-L-ascorbic acid trisodium salt (VitC) (Sigma #49752-10G); d7: DMEM containing 1:100 SM1, 1:100 NEAA, 1mM Sodium Pyruvate, 1:100 GlutaMAX, 3nM TTNPB, and 50 μg/ml VitC; d8-9: DMEM containing 1:100 SM1, 1:100 NEAA, 1mM Sodium Pyruvate, 1:100 GlutaMAX, 100 ng/ml EGF (R&D Systems #236-EG-01M), 50 ng/ml KGF, and 50 μg/ml VitC; d10-16: DMEM containing 2% fraction V BSA, 1:100 NEAA, 1mM Sodium Pyruvate, 1:100 GlutaMAX, 1:100 ITS, 10ug/ml Heparin (Sigma #H3149-250KU), 2 mM N-Acetyl-L-cysteine (Cysteine) (Sigma #A9165-25G), 10 μM Zinc sulfate heptahydrate (Zinc) (Sigma #Z0251-100g), 1x BME, 10 μM Alk5i II RepSox (R&D Systems #3742/50), 1 μM 3,3’,5-Triiodo-L-thyronine sodium salt (T3) (Sigma #T6397), 0.5 μM LDN, 1 μM Gamma Secretase Inhibitor XX (XXi) (AsisChem #ASIS-0149) and 1:250 1M NaOH to adjust pH to ~7.4; d17-23: CMRL (Gibco #11530-037) containing 1% BSA, 1:100 NEAA, 1 mM Sodium Pyruvate, 1:100 GlutaMAX, 10ug/ml Heparin, 2mM Cysteine, 10uM Zinc, 1x BME, 10 μM Alk5i II RepSox, 1 μM T3, 50 μg/ml VitC, and 1:250 NaOH to adjust pH to ~7.4. All medias also contained 1x PenStrep.

### Flow cytometry

Single cell sBC were filtered through cell strainer into FACS 5ml tubes and incubated for 30 min on ice for surface markers or overnight at 4°C for intracellular markers. After incubation, the cells were washed and strained again through cell strainer and resuspended in FACS buffer for analyses on CYTEK Aurora. Analysis and graphs were made using FlowJo software v10.6.2.

### CRISPR-Cas9 and TALEN mediated genome engineering

Generation of hPSC lines has been described previously (34). Briefly, hPSC Mel1 ^INS-GFP^ cells were dissociated into single cells using TrypLE incubation at 37°C for 8 min. Cells were then quenched with mTeSR+ media and counted using MoxiGo II cell counter. 2 x 10^6^ cells were transferred into microcentrifuge tubes and washed twice with PBS. Washed cells were then prepared for nucleofection of TALEN mediated knock-in (KI) of a Tet-On PD-L1 inducible system (i) or a CRISPR-Cas9 mediated B2M gene knock-out (ii). Cells were nucleofected in P3 buffer following the Amaxa P3 Primary cell 4D-Nucleofector kit protocol (V4XP-3024) using the CB-150 program. (i) AAVS1-TALEN-L and AAVS1-TALEN-R (gift from Danwei Huangfu, Addgene plasmid # 59025; http://n2t.net/addgene:59025; RRID: Addgene 59025) as well as a Tet-On inducible PD-L1 overexpression plasmid (generated in house) were nucleofected into Mel1 ^INS-GFP^ hPSC. Nucleofected cells were then plated in 10 cm plates with 10 μM ROCK inhibitor. After 48 hr of plating, puromycin selection (0.5 μg/ml) was performed for 48 hr then removed for 48hr followed by neomycin selection (50 μg/ml) for 6 days. Remaining colonies were picked and amplified for further characterization. (ii) guide RNAs (gRNA) targeting exon 1 and exon 2 of the B2M gene were generated in house and nucleofected along with CRISPR-Cas9. 24hr after plating, cells were selected for 48 hr with puromycin (0.5 μg/ml). Single colonies were picked and amplified for further characterization. Genomic DNA was extracted from targeted colonies and PCR analysis for TALEN KI and B2M KO was performed to identify potential modified clones. Further Sanger sequencing of PCR products confirm B2M mutations in the genome of Mel1 ^INS-GFP^ hPSC.

### Autoimmune diabetes model

The mouse strain NOD-cMHCI^-/–^A2 (Jax Stock No: 031856) was provided by Dr. Dave Serreze at The Jackson Laboratory. Mice were maintained in University of Florida Institutional Animal Care Services and genotype was confirmed by PCR using a commercial service (Transnetyx). All experiments involving animals were approved by the University of Florida Animal Care and Use Committee (IACUC no. 201810300). NOD-cMHCI^-/–^A2 mice underwent transplant of 1,000 sBCs under the left kidney capsule, similarly to transplantation of pancreatic islets as described (5, 8, 36). Mice were anesthetized with 1.5-2% isoflurane, shaved and cleaned with alternate washes of sterile saline and 2% chlorhexidine. Subcutaneous injection of buprenorphine was administered as post-operative analgesic. Four days prior to transplant, mice were either kept in a standard feed diet (Envigo 2918) or changed to a doxycycline (DOX) infused feed diet (Envigo TD.01306) *ad libitum* for the remainder of the study. The animals weight was recorded the day of surgery and every week after that.

Diabetes was confirmed by blood glucose > 250 mg/dl on two consecutive measurements by tail tip blood with ACCU-CHEK^®^ Guide glucose meter. Mice with blood glucose >400 mg/dl were administered with 1-2s unit of Lantus^®^ long-acting insulin, daily. The age of the mice in the study ranged from 15 weeks to 29 weeks. Two weeks after transplantation the graft-bearing kidneys and mice pancreas were recovered for immunohistochemistry analysis.

### *In vitro* bioluminescence by sBCs

The iP-sBCs were genetically engineered to express constitutive firefly luciferase for longitudinal monitoring after transplantation (34). sBCs bioluminescence was tested *in vitro* in a 96-well polystyrene black plate (Fisher no. 12-566-620) at different densities (0, 25, 50 or 100 clusters). iP and iP + DOX sBCs were incubated for 5 minutes at 37°C in media with or without D-luciferin (150 µg/mL) supplementation, prior to bioluminescence imaging. Bioluminescence was detected with an IVIS^®^ Spectrum *In Vivo* Imaging System (IVIS, PerkinElmer, Waltham, MA, USA). For bioluminescence imaging, the system was set to take images without any emission filter (open) and chamber temperature set to 37°C.

### Longitudinal monitoring of the sBCs grafts by transdermal bioluminescence

Following transplantation, the sBC grafts’ viability was longitudinally monitored by transdermal bioluminescence. A fresh stock solution of D-luciferin (PerkinElmer no. 122799) was prepared at a concentration of 30 mg/mL and filtered through a 0.22μm Spin-X^®^ centrifuge tube filter (Costar no. 8160) at each intervention. Mice were anesthetized with 1.5-2% isoflurane and sterile ocular lubricant was applied. Hair on the left lateral side of the mice was removed with depilatory cream. D-Luciferin was injected subcutaneously at 75 mg/kg five minutes prior to imaging. Transdermal bioluminescence was captured with an IVIS^®^ Spectrum *In Vivo* Imaging System. For bioluminescence imaging, the system was set to take images without any emission filter (open) and sequential imaging was performed until signal peaked. Transplants were longitudinally monitored at days 1, 3, 7 or 9, and 14 after surgery.

*In vivo* bioluminescence imaging data was analyzed using PerkinElmer, Inc Living Image^®^ 4.5.5 software. The total flux (p/s) was estimated with a square region of interest (ROI) over the left kidney region. A corrected total flux (CTF) was determined by measuring the background signal in the leg and applying the following formula:


$$CTF=\text{Total flux at kidney}–(\text{background reading}*\frac{ROI\;area\;at\;kidney}{ROI\;area\;of\;background})$$

### Immunohistochemistry

Graft-bearing kidneys and pancreas were fixed with 3.2% paraformaldehyde (Thermo Fisher no. 047377) at 4°C in PBS overnight followed by washes with PBS. The tissues were immersed in 15% w/v sucrose in PBS overnight, followed by 30% w/v sucrose overnight then snap frozen in Optimal Cutting Temperature (O.C.T) compound (Thermo Fisher) using 2-methylbutane (>99% purity) chilled with liquid nitrogen. Frozen tissue blocks were cut with a cryostat into serial 10 μm sections and mounted on Superfrost Plus microscope slides (Thermo Fisher).

Cryosections were incubated twice for 15 minutes in 0.3% Triton X-100. Samples were then blocked and permeabilized in PBS with 0.3% Triton X-100 + 10% donkey serum for 1 hour at room temperature. Primary antibodies were incubated overnight in PBS with 0.3% Triton X-100 + 1% donkey serum at 4°C. CF488A-, CF568A- and 650 Dylight-conjugated secondary antibodies were incubated at 1:200 dilutions in PBS with 0.3% Triton X-100 for 1 hour at room temperature. Coverslips were mounted with ProLong™ Gold with DAPI (Thermo Fisher).

### Antibodies

The following primary antibodies were used for immunofluorescence staining with their respective dilution: Guinea pig-anti-insulin (Dako no. A0564, 1:1000), Rat-anti-mouse CD3 (Biolegend no. 100209, 1:50), Rabbit-anti-human PD-L1 (Abcam no. ab205921, 1:200), Rat-anti-mouse CD45 (Sigma Aldrich no. 05-1416, 1:100), Rat-anti-mouse FOXP3 (Biolegend no. 126401, 1:150), Goat-anti-mouse CD4 (Fisher no. AF554SP, 1:200) and Rabbit-anti-laminin (Abcam no. ab11575, 1:300). CF, Alexa and Dylight secondary antibodies were acquired from Sigma-Aldrich and Invitrogen, respectively.

### Analysis of the histochemical data

Histochemical samples of the graft-bearing kidneys were analyzed for the number of insulin positive cells (Ins^+^ cells), PD-L1^+^ and CD3^+^ cells. The whole graft section in the kidneys was imaged by tile scanning on a Leica SP8 Confocal microscope with a 20x/0.75 numerical aperture Plan-Apochromat air objective.

All image samples were analyzed using Fiji software (ImageJ). Prior to analysis, a max intensity projection (MIP) of the z-stack was generated. Image channels were split and the CD3^+^ channel was subtracted from the Ins^+^ channel with the image calculator. PD-L1^+^ channel was binarized with threshold set automatically using the moments model. An ROI (ROI-1) was hand drawn around the inner graft area using the CD3^+^ channel as reference. The number of Ins^+^ and PD-L1^+^ cells per unit area in ROI-1 was measured using the Fiji Plug-In StarDist 2D with the following settings: [Model: Versatile (fluorescence nuclei); Normalize Image; Percentile (1.0 - 99.8); Probability/Score (0.7); Overlap (0.4)] (37). Manual counting was used if the number of cells was less than 15 total to avoid a StarDist error.

A second ROI (ROI-2) was hand drawn on the CD3^+^ channel around the outer graft area. The number of CD3^+^ cells per unit area in ROI-2 was determined using StarDist 2D with the following settings: [Model: Versatile (fluorescence nuclei); Normalize Image; Percentile (1.0 - 99.8); Probability/Score (0.6); Overlap (0.4)] (37).

For this analysis 3 to 4 MIPs tile scans of 10 μm thin sections of 2 to 4 recovered graft-bearing kidneys for each group, from which graft was successfully located, were utilized. The inner graft area of the histochemical samples was determined for the analysis as the immediate area enclosing all the insulin positive cells. While, the outer graft area was determined as the area encompassing the whole graft adjacent to the kidney tissue wall minus the inner graft area. All images were processed in the same way.

### Statistical analysis

One-Way ANOVA with multiple comparisons was used to determine significant changes for the flow cytometry analysis. IVIS Imaging bioluminescence data was analyzed by Two-Way ANOVA. Repeated measures from the same mouse were matched by time point and corrected with Geisser-Greenhouse correction method (if ϵ < 0.75). For immunohistochemistry quantification, means between two groups were compared by two tailed Student’s t-test. A confidence level of 95% was considered significant. The statistical test used, P values and definition of n are indicated in the individual figure legends. Error bars display the standard error from the mean (± s.e.m). Statistical analyses were performed in GraphPad Prism 9.3 software.

## Results

### Characterization of sBCs with inducible PD-L1 system and HLA class I KO

Programmed death-1 (PD-1) is an inhibitory receptor expressed on the surface of antigen-stimulated T cells that is important for maintenance of tolerance (38). The ligand of PD-1, PD-L1, is expressed on a variety of somatic cell types and is important for regulating the immune system’s response to self-antigen by down-regulating T cell inflammatory activity. Binding of PD-L1 to its receptor PD-1 on activated T cells inhibits T cell activation signals and inhibits CD8^+^ T cell cytotoxicity. In the context of autoimmune diabetes, systemically disrupting PD-1/PD-L1 interactions with a monoclonal antibody or through genetic deficiency accelerates T1D in NOD mice (39). PD-1/PD L1 checkpoint inhibitors used clinically to treat cancer in humans can result in autoimmune side effects including an aggressive form of autoimmune diabetes termed fulminant T1D (40, 41). Thus, augmenting PD-1/PD-L1 signaling in the local vicinity of the islet is a valid strategy to counter islet immune rejection (23).

**Figure 1:**
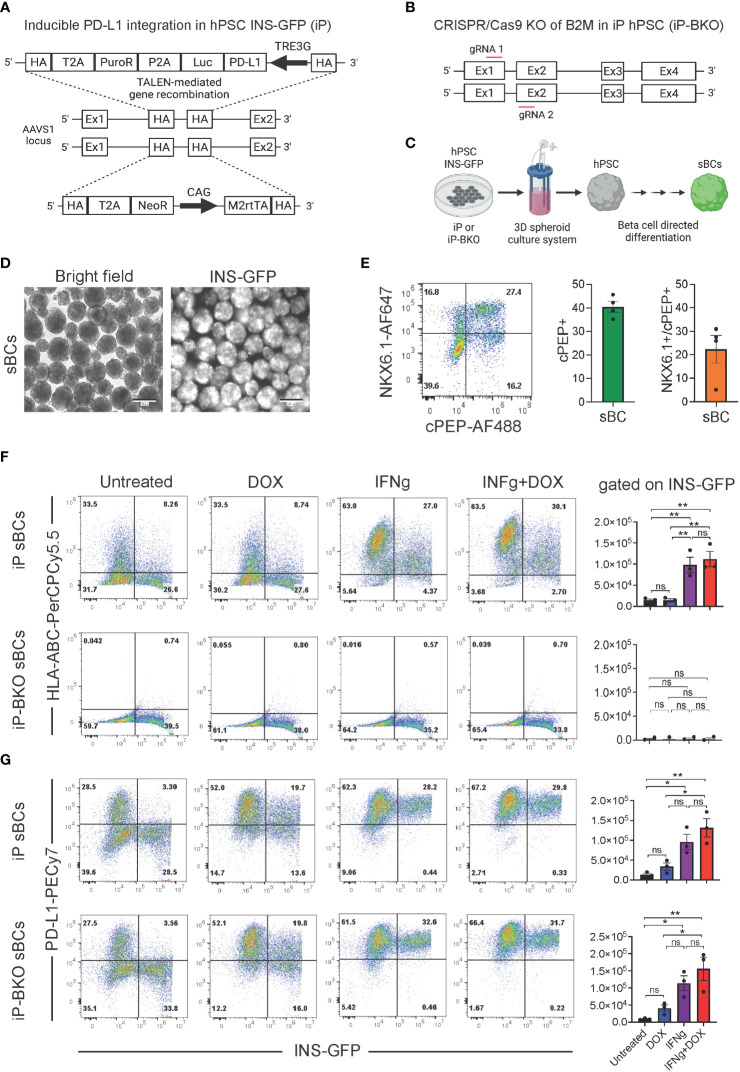
Quantification of HLA class I and PD-L1 expression on stem cell derived beta cells containing inducible PD-L1 expression with or without B2M knock out. **(A)** Schematic of the PD-L1 inducible system integrated into the AAVS1 locus of human pluripotent stem cells that contain a GFP fluorescence reporter under the control of the endogenous insulin promotor (INS-GFP). Constitutive expression of a puromycin resistance, a luciferase gene and a neomycin resistance gene is driven by the endogenous AAVS1 gene *via* T2A peptide cleavage sites on either allele, respectively. The transgene cassette also contains a strong CAG promoter driving constitutive expression of the M2rtTA that facilitates DOX inducible expression of PD-L1 under the control of the TRE3G promoter, bicistronicly. **(B)** Schematic of the CRISPR/Cas9 knock-out (KO) of B2M gene. Two gRNAs directed towards exon 1 and exon 2 of the B2M gene were used resulting in B2M gene KO and inhibition of HLA class I surface expression. **(C)** Schematic of the 3D suspension based directed differentiation protocol for the generation of stem cell derived beta cells (sBC) from human pluripotent stem cells (hPSC). **(D)** Bright field and fluorescence images of differentiated sBCs containing an endogenous insulin promoter driven GFP (INS-GFP) reporter. Scale bar represents 200 µm. **(E)** Representative flow cytometry analysis (left) and associated quantification (right) for beta cell markers cPEP and NKX6.1 of differentiated sBCs. Single positive cPEP and double positive cPEP and NKX6.1 cells are quantified. n = 4 independent experiments employing both iP and iP-BKO cells. **(F)** Representative HLA-ABC and INS-GFP flow cytometry analysis and associated quantification of differentiated iP sBC- (top row) and iP-BKO sBC- clusters (bottom row) untreated or treated with DOX (2 ug/ml), IFNg (100 ng/ml) or both for 48 hr. Bar plots represent HLA-ABC MFI quantification of INS-GFP+ cells from n = 3 independent differentiation experiments. One-Way ANOVA with multiple comparisons was used to determine significant changes between the groups (n = 3 for iP-BKO). **(G)** Representative PD-L1 and INS-GFP flow cytometry analysis and associated quantification of differentiated iP sBC- (top row) and iP-BKO sBC- clusters (bottom row) untreated or treated with DOX (2 ug/ml), IFNg (100 ng/ml) or both for 48 hr. Bar plots represent PD-L1 MFI quantification of INS-GFP+ cells of n = 3 independent differentiation experiments. One-Way ANOVA with multiple comparisons was used to determine significant changes between the groups (n=3 for iP-BKO). All error bars represent mean ± s.e.m. * p < 0.05 and ** p < 0.01. ns, Not significant.

We previously evaluated the protective capability of sBCs with overexpression of PD-L1 and deletion of HLA surface expression to counteract autoreactive CD8^+^ TCR stimulation *in vitro* (34). Similarly, we used the PD-L1 inducible system that was integrated into the AAVS1 locus of hPSC that contains a GFP fluorescence reporter under the control of the endogenous insulin promotor (INS-GFP). The transgene cassette also contains a strong CAG promoter driving constitutive expression of the M2rtTA that facilitates the Tet-On–DOX inducible PD-L1 expression system (referred to as iP) under the control of the TRE3G promoter. Site specific integration was possible using transcription activator-like effector nucleases (TALEN) technology (**Figure 1A**). HLA class I surface expression by sBCs was disrupted by knockout (KO) of the beta-2- microglobulin (B2M) gene (referred to as iP-BKO) with CRISPR/Cas9 by two gRNAs directed towards exon 1 and exon 2 of the B2M gene (**Figure 1B**). sBCs were generated from iP and iP-BKO hPSCs employing a 3D suspension based directed differentiation protocol, as previously described (34), and INS-GFP expression was confirmed by live fluorescence imaging (**Figures 1C, D**). After completion of the differentiation protocol, dual-expression of key beta cell markers C-peptide (cPEP, a byproduct of endogenous insulin biosynthesis) and NKX6.1 was determined by flow cytometry (**Figure 1E**). About 40% of total cells expressed cPEP at the end of the differentiation protocol. Furthermore, the percentage of NKX6.1 and cPEP double positive cells is ~25% on average for both lines. Introduction of the PD-L1 inducible system or deletion of all HLA class I surface molecules *via* beta-2 microglobulin knockout was previously demonstrated to not decrease or impede insulin expression (34). Efficiency of the PD-L1 DOX inducible system and ablation of HLA class I surface expression (BKO) was determined by flow cytometry analysis on iP and iP-BKO sBCs from independent differentiation experiments treated with exogenous DOX and/or interferon-γ (IFNg) cytokine (**Figures 1F, G**). IFNg but not DOX treatment increased HLA class I expression in iP sBCs, while iP-BKO sBCs had essentially no HLA Class I expression in all treatment groups. This data confirms that sBCs upregulate HLA class I when exposed to inflammatory cytokines and complete ablation of HLA class I molecules in iP-BKO sBCs was successful. DOX and IFNg treatments individually increased PD-L1 expression in iP and iP-BKO sBCs. Moreover, combination of both treatments show the highest PD-L1 induction in both iP and iP-BKO sBCs. Taken together, we show quantitative PD-L1 and HLA class I surface expression on sBC with inducible PD-L1 expression in the presence or absence of HLA class I molecules under distinct disease modeling conditions.

### *In vivo* surveillance of transplanted sBCs

To quantify and assess the viability of sBC grafts post-transplantation we utilized the constitutively expressed bioluminescence reporter luciferase (**Figure 1A**). Luciferase is a well-characterized reporter protein commonly used to monitor and quantify tumor growth or cell populations over time *in vivo*. When luciferase oxidizes its substrate, luciferin, it results in light emission that can be detected with a camera (42). Prior to transplantation, sBC bioluminescence was confirmed *in vitro* by incubating sBC clusters at different densities with D-luciferin and detected with an IVIS^®^ Spectrum *In Vivo* Imaging System. Wells that had no sBCs or were not supplied with D-luciferin did not emit any bioluminescence, as expected (**Supplementary Figure 1**). Results show that total flux is directly proportional to the number of clusters (**Supplementary Figure 1**).

The mouse strain NOD-cMHCI^-/–^A2 was selected for this study because it develops spontaneous autoimmune diabetes and recapitulates human HLA Class I (24). Due to the HLA-A2 match, human sBC grafts should therefore experience autoimmune pressure from immune recognition of islet antigens but not invoke an immediate xenograft response. A limitation of the model is that NOD-cMHCI^-/–^A2 mice could still develop adaptive immunity toward other human antigens including non-classical HLA molecules. However, immune tolerance strategies by others have been shown to be effective at controlling xenoantigen responses in non-autoimmune beta cell transplant settings (23).

**Figure 2:**
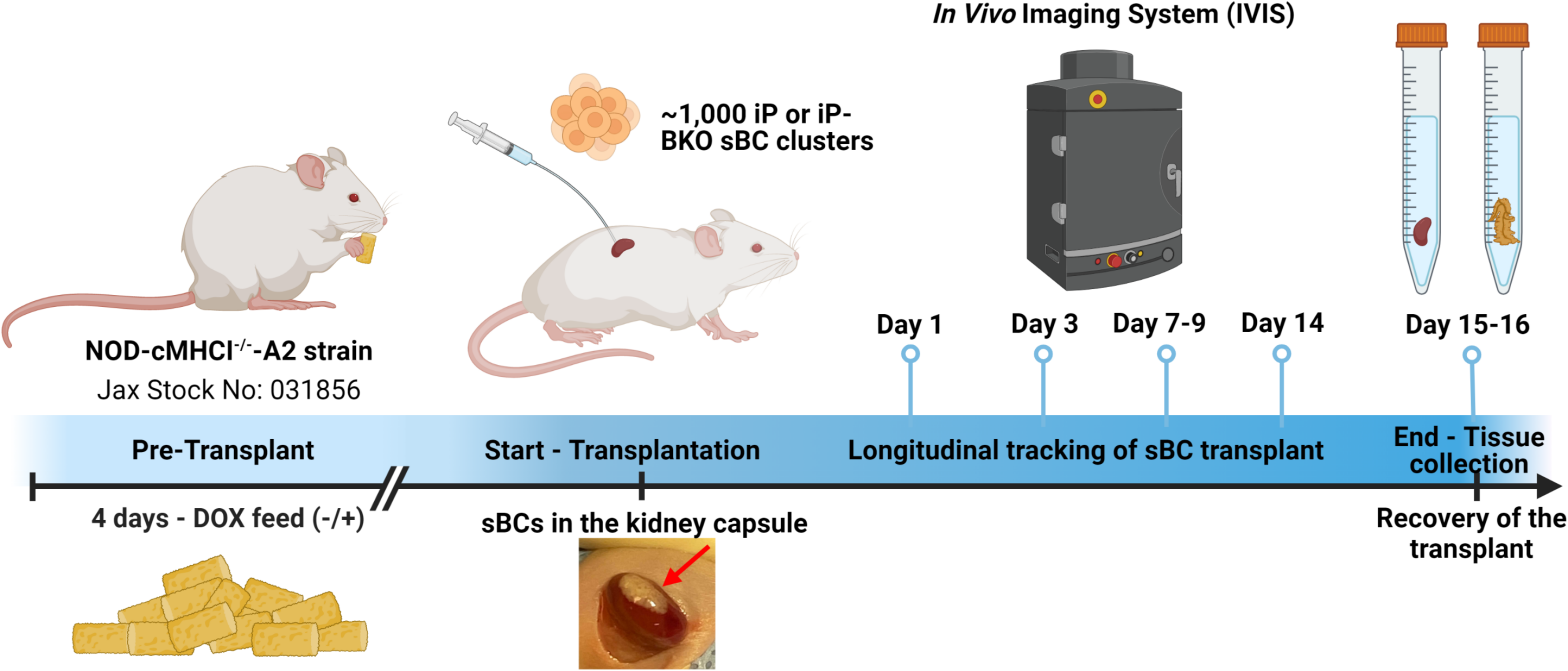
Timeline of the study. HLA-A2 and BKO sBC clusters were generated with a Tet-On PD-L1 inducible system (iP) and constitutive luciferase expression. PD-L1 is expressed by the sBC clusters in presence of doxycyline (DOX). Four days prior to transplantation, mice were put on a special diet with doxycycline (+) or maintained on standard diet (-). About 1,000 sBC clusters were transplanted to the left kidney capsule of each mouse. Survival of the transplanted sBCs was monitored by transdermal luminescence detection with IVIS. The sBC graft-bearing kidneys were recovered after completion of the 2-week study period for histology. Created with Biorender.com.

**Figure 3:**
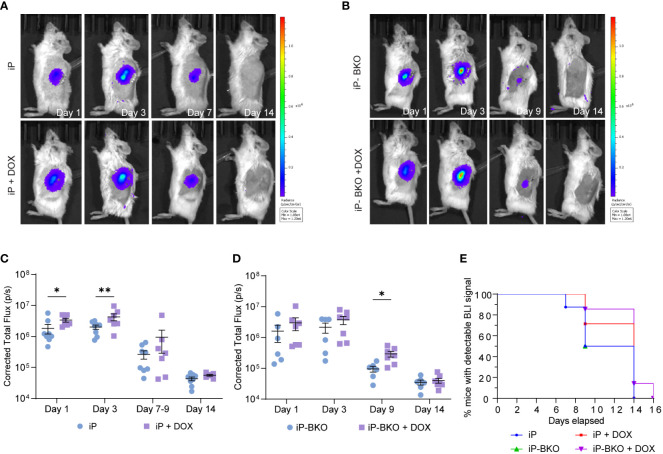
Longitudinal monitoring of the sBC graft by transdermal bioluminescence detection *via* IVIS. **(A, B)** Representative images of bioluminescence expression by **(A)** iP-sBC or **(B)** iP-BKO sBC graft after transplant on day 1, 3, 7 or 9, and 14. **(C, D)** Quantification of the total flux measured from mice of both sex in the **(C)** iP-sBC and **(D)** iP-BKO sBC experiments. Each sBC group is subdivided by +/- DOX treatment as follows: iP (n=7), iP + DOX (n=8), iP-BKO (n=6) and iP-BKO + DOX (n=7). Two-Way ANOVA, *p < 0.05, **p-value < 0.01 mean ± s.e.m. **(E)** Kaplan-Meier curve for percentage of mice with detectable bioluminescence signal for all groups.

One thousand iP or iP-BKO sBC clusters with or without DOX pretreatment in the culture medium were transplanted under the kidney capsule of NOD-cMHCI^-/–^A2 mice. Mice in the +DOX group were switched to a DOX infused feed diet four days prior to surgical intervention (**Figure 2**). The viability of the transplanted sBC grafts under the kidney capsule of mice for experiments was monitored by transdermal bioluminescence for a period of two weeks with IVIS Imaging System (**Figure 2**). D-luciferin was administered to the mice subcutaneously, then sequential imaging was performed until bioluminescence signal peaked (**Figures 3A, B**). Animal weight was monitored throughout the study and showed no significant differences between experimental groups (**Supplementary Figure 2**).

A two-way ANOVA of the total photon flux detected longitudinally by IVIS showed a statistical difference (p-value <0.05) for mice transplanted with iP sBCs + DOX feed at day 1 and 3 (**Figure 3C**). The data suggest that sBCs expressing PD-L1 were more resistant to immune rejection in the first 3 days, but that this expression was insufficient for full graft survival. Results from the iP-BKO + DOX sBCs showed a significant improvement in beta cell survival later, at day 9 (**Figure 3D**). Because the differences in survival with PD-L1 expression at the earlier day 3 timepoint were not seen for BKO sBCs, we speculate that the initial mechanism of immune attack is likely to be predominantly HLA- class I driven. When comparing all iP and iP-BKO sBC, mice transplanted with iP-BKO + DOX had the highest number of animals with detectable bioluminescence at late time points (**Figure 3E**). Although luciferase signal dropped below detection limits by day 14 for most mice, one mouse from the iP-BKO + DOX group showed detectable transdermal luminescence at day 14 (**Supplementary Figure 3**). In addition, IVIS scan of the kidney removed *ex vivo* at day 16 showed surviving sBC clusters.

### PD-L1 overexpression and HLA Class I KO improve sBC survival

**Figure 4:**
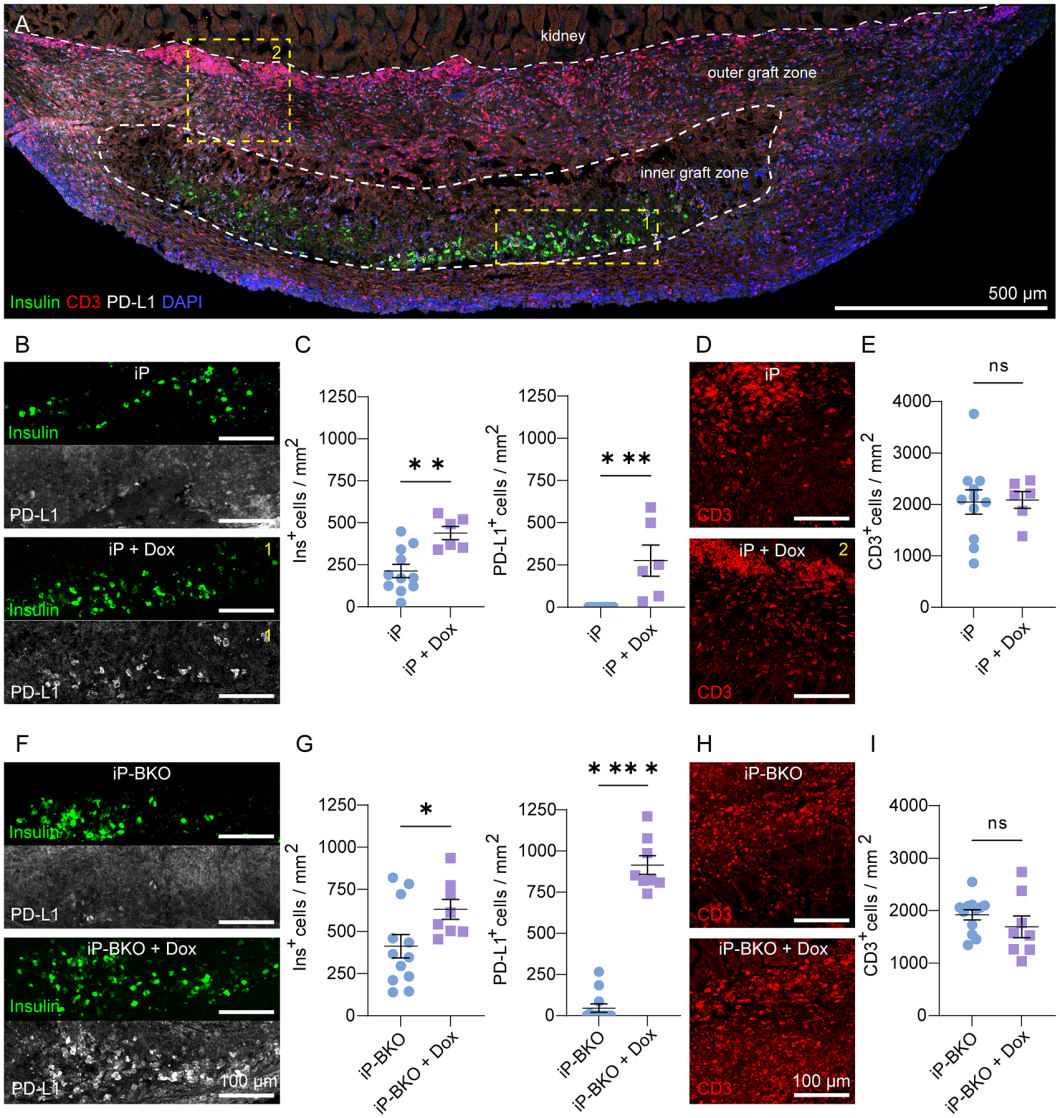
Insulin and PD-L1 expression by sBCs 2-weeks after transplantation. **(A)** Representative maximum intensity projection (MIP) image of whole graft-bearing kidney tissue section from the iP-sBC +DOX group immunostained for insulin, PD-L1 and CD3. **(B, C)** Expression and quantification of insulin and PD-L1 by iP and iP + DOX sBCs in inner graft zone. **(D, E)** Representative images and quantification of CD3^+^ cells in the outer graft zone of iP and iP + DOX sBCs. **(F, G)** Expression and quantification of insulin and PD-L1 by iP-BKO and iP-BKO + DOX sBCs in inner graft zone. **(H, I)** Representative images and quantification of CD3^+^ cells in the outer graft zone of iP-BKO and iP-BKO +DOX sBCs. The inner and outer graft zone in **(A)** are indicated with white dashed line and the regions enclosed in yellow dashed line depict location in the graft-bearing kidney from which the high magnification images shown of insulin and PD-L1 **(B)** or CD3 **(D)** were taken. n = 8 to 12 immunostained 10 μm thin sections of 2 to 4 recovered graft-bearing kidneys per condition, from which graft was successfully located. Two-tailed Student’s t-test, *p = 0.0394, **p = 0.0023, ***p = 0.0008, ****p < 0.0001, mean ± s.e.m. ns, Not significant.

To further elucidate the effects of PD-L1 overexpression and HLA Class I KO on the survival of the transplanted sBCs we performed detailed immunohistochemistry on the recovered graft-bearing kidneys. Cryosections sections of the graft area were stained for insulin, CD3, CD45, CD4, FOXP3 and PD-L1. After two weeks *in vivo*, all groups had significant numbers of surviving insulin^+^ cells in the inner graft region, surrounded by a robust immune infiltration of CD3^+^ (**Figure 4A**; **Supplementary Figure 4**), CD45^+^ cells (**Supplementary Figure 5**) and CD4^+^ (**Supplementary Figure 6**). Large numbers of CD3^+^ cells accumulated at the interface between the kidney tissue and the graft site and lower numbers of CD3^+^ cells were observed in the inner graft zone near sBCs. No qualitative differences were observed in the localization of CD3^+^ cells between any of the groups. However, some of our histology samples possesses a bright red autofluorescence near the center of the graft, in the same channel we used for CD3 staining. We believe this autofluorescence stems from cellular debris, as it lacks a cellular morphology and can be observed in unstained control slides (**Supplementary Figure 7**). Mice were found to be experiencing severe insulitis in the pancreas during the same timeframe as the sBC graft experiment, indicating an active autoimmune response to beta cell antigens (**Supplementary Figure 8**).

In the first experiment with transplantation of sBCs expressing HLA-A2, DOX treatment doubled the number of surviving insulin^+^ cells in the inner graft region with an average of 213 ± 39 and 438 ± 39 insulin^+^ cells/mm^2^ ± s.e.m. for iP and iP + DOX, respectively (**Figures 4B, C**). DOX-inducible expression of PD-L1 was extremely robust with no PD-L1^+^ cells detected in the graft region for iP and 275 ± 92 PD-L1^+^ cells/mm^2^ ± s.e.m for iP + DOX. There was no difference in the number of CD3^+^ cells between the two groups (**Figures 4D, E**), indicating that DOX treatment and PD-L1 expression did not alter the number of T cells at the time point examined. A reduction in T cell number is not expected because PD-L1 works by inhibiting T cell activation and does not induce T cell death as its name might suggest (38, 43). *In vitro*, IFNg treatment induces sBC PD-L1 expression (**Figure 1**). Thus, the lack of PD-L1 expression *in vivo* in the absence of DOX suggests lower levels of IFNg in the local immune environment. The number of PD-L1 expressing cells was 60% of the number of insulin-expressing cells, consistent with the PD-L1 expression trends observed *in vitro* (**Figure 1**). The HLA-A2 matching in this model likely enabled the partial survival of insulin^+^/PD-L1^-^ sBCs, while DOX-induced PD-L1 increased the number of surviving cells.

In the second experiment with transplantation of BKO sBCs that lack expression of HLA Class-I, the number of surviving insulin^+^ cells increased to 412 ± 69 and 631 ± 59 insulin^+^ cells/mm^2^ ± s.e.m for iP-BKO and iP-BKO + DOX, respectively (**Figures 4F, G**). The increased number of insulin^+^ cells in both groups relative to the first experiment suggests the lack of MHC-I expression may improve sBC survival rates. As before, DOX treatment significantly improved sBC survival and robustly induced PD-L1 expression from 46 ± 25 to 915 ± 57 PD-L1^+^ cells/mm^2^ ± s.e.m for iP-BKO and iP-BKO + DOX, respectively. Higher magnification of the graft better demonstrate insulin^+^ cells co-expressing PD-L1 (**Supplementary Figure 9**), consistent with the *in vitro* data (**Figure 1**). Differently from the first experiment, in the iP-BKO sBCs DOX treatment resulted in a slighter greater number of PD-L1-expressing cells than insulin-expressing cells. There was also a non-zero mean number of PD-L1 expression for the group without DOX. These results suggest a slightly more inflammatory environment (i.e. higher IFNg) in the iP-BKO grafts. The total number of infiltrating CD3^+^ cells remained the same as the previous experiment and was not significantly different for +/- DOX in either experiment (**Figures 4H, I**).

To better characterize the protective ability imparted by PD-L1 overexpression on the transplanted sBCs the presence of regulatory T (Tregs) cells was investigated. Activation of PD-1/PD-L1 pathway play an important role in the promotion of Tregs development and peripheral tolerance (Francisco LM et al., 2009; Fanelli G et al., 2021). Qualitative analysis of the processed samples suggests that PD-L1 overexpression by iP + DOX and iP-BKO + DOX samples resulted in a greater number of FOXP3^+^ cells (**Supplementary Figure 6**). The FOXP3^+^ cells were primarily found in the areas where abundant CD4^+^ cells are present. These finding suggest that the increased survival of sBCs in the iP + DOX and iP-BKO + DOX can be partly attributed to development and recruitment of Tregs at the graft site.

Additionally, in the interest of assessing the surviving sBCs capacity to regulate glycemia, blood glucose was monitored throughout the study and blood serum was collected from some of the mice at the study end point to evaluate human C-peptide levels. Grafted iP-sBCs were unable to reverse hyperglycemia within the period of the study. Human C-peptide ELISA showed detectable human C-peptide in a small number of mice at the study end point, albeit at low levels. We did not add this data to the manuscript as it was not routinely measured at all timepoints or for all mice, as our study was focused on the immune rejection of the sBCs. Moving forward, a different dosage of iP-sBCs would be evaluated to determine the amount required to have a significant effect on blood glucose in this model.

## Discussion

This study focused on transplantation of stem cell-derived beta-like cells in a mouse model with spontaneous diabetes and matching human HLA class I-A2. To our knowledge this is the first time a study demonstrates robust recruitment of T-cells at the graft site concurrent with substantial autoimmune insulitis in the pancreas in a pseudo-allogenic human/mouse model. Immunohistochemistry showed substantial insulitis in the mouse pancreas indicating a background of strong islet-directed autoimmunity. Diabetogenic CD8^+^ T cells from NOD-cMHCI^-/–^A2 mice can target and kill human beta cells from A2 islet donors in an antigen-specific manner (24). We utilized HLA-A2 sBCs with a Tet-On system to tightly control PD-L1 expression *in vivo* with DOX treatment. Inducible expression of PD-L1 improved the survival of sBCs in the immunocompetent HLA-A2 NOD mice. Additionally, we showed that knockout of HLA Class I by disrupting B2M further improved survival of the sBC grafts and was further enhanced by PD-L1 expression.

sBCs carry tremendous potential as an abundant source of functional beta cells for cell replacement therapy. Ongoing and planned clinical trials using stem cell derived pancreatic cells rely on cell encapsulation and/or systematic immune suppression, both of which have their own sets of challenges. Immediate and sustained local immune suppression could provide distinct advantages for current cell therapy efforts. Thus, it is important to explore approaches that can provide localized protection to sBC grafts *in vivo*. However, despite great research efforts focused on developing and utilizing *in vivo* models for testing critical aspects of sBC cell therapy, humanized models that accurately recapitulate autoreactive TCR-HLA immune-/beta cell- interactions *in vivo* are largely absent. In contrast, our approach allows us to investigate the immunosuppressive capacity of modifications to sBCs such as surface expression of PD-L1 in a human TCR-peptide-HLA dependent manner *in vivo*, as shown here. We anticipate that our proposed experiments represent a critical first, but absolutely necessary, step towards interrogating immune-beta cell interactions in an autologous and autoimmune manner, directly providing key advancements beyond previous studies that enabled allo- or xenogenic immune investigations only.

Development of mouse models with human immune cell engraftment, often referred to as humanized mice or human immune system mice, have been a major component of testing strategies for human sBC pseudo islet tolerance. Humanized mice usually involve a severely immunodeficient host, for example the NSG (NOD scid gamma mouse) that has been reconstituted with allogenic human immune cells. Humanized mouse models allow for the *in vivo* study of T cell modular immune therapeutics, but also have several limitations (44) for their utility to simulate human insulitis and T1D. NSG mice develop graft versus host disease when reconstituted with a human immune system through recognition of the mouse xenoantigens by the transferred human immune cells (45). This race against development of graft versus host diseases imposes an artificial time limit on the experiment duration, although this limitation can be ameliorated though the use of MHC-I/II knockout NSG mice (46). Humanized mice used for islet transplantation also examine mechanisms of allo-rejection and these models do not have an autoimmune component with the exception of a few examples where immune cells were transferred from patients with T1D (47) or represented a T cell clone with an islet-specific TCR (48, 49). Clinical human islet replacement therapy is challenged by both concurrent autoimmune and alloimmune rejection. Humanized mouse models typically ignore autoimmunity as a variable in transplant tolerance studies. Instead, diabetes is usually induced in these animals through streptozotocin (STZ) injection and only allo-immunity is studied (50). This is a problem for clinical translation because tolerance strategies that successfully induce allo-tolerance do not provide clear information on feasibility for inducing autoimmune tolerance.

A further limitation of humanized mice is that the adoptively transferred human immune cells do not traffic effectively to the islet graft site. Thus, transplanted islets must be injected directly into the spleen to increase encounters with T cells (51–53). Our approach makes steps to address this key challenge in the field by demonstrating robust T cell trafficking to the graft and enabling the use of the kidney capsule as a viable transplant site for studies of human sBC tolerance.

Our data show that PD-L1 overexpression by the engineered sBCs reduced the severity of immune rejection in the first 3 days for HLA-A2 expressing sBCs. In BKO sBCs, PD-L1 overexpression differentially affected the graft survival later, at day 9. One mouse in the iP-BKO + DOX group showed surviving human cells detectable by IVIS at day 14. Histological examination revealed significant numbers of surviving insulin^+^ and PD-L1^+^ sBC cells despite extensive infiltration by CD3^+^, CD4^+^ and CD45^+^ cells. The timing of rejection and extensive graft site immune infiltration suggests that immune responses compromised the sBC grafts at early time points but that significant numbers of insulin and PD-L1 expressing cells managed to survive in this immunological environment. Expression of PD-L1 *in vivo* was tightly controlled by DOX and significantly improved sBC survival as measured by IVIS and histology. However, others were more successful with human islet-like organoids (HILOs) modified to overexpress PD-L1 and prevented xeno-rejection for 50 days in immunocompetent mice (23). Nevertheless this strategy has yet to be tested in a model with islet-directed autoimmunity. Our data suggest that PD-L1 alone may not be sufficient to prevent rejection in a setting with spontaneous autoimmunity.

In our model, deletion of HLA Class-I resulted in approximately twice as many remaining sBC over HLA-A2 expressing sBC for both +/- DOX groups, suggesting that MHC-I knockout is a valid strategy for improved autoimmune tolerance in beta cell replacement therapy. However, it remains that even with HLA Class I KO and PD-L1 overexpression sBCs are unable to fully evade the immune system in this model combining autoreactive T-cells and subsequent xenoresponse. Our results are complementary to other studies showing that MHC-I knockout prolongs survival of syngeneic and allogenic mouse islets when transplanted into diabetic NOD mice (54–56). Additionally, others have shown with more success that selective retention of HLA-A2 but knockdown of other HLA Class IA molecules was able to promote beta cell graft survival for up to 8 weeks from transferred allogenic but HLA-A2 matched human PBMCs (13). In T1D, beta cells under an inflammatory environment upregulate expression of HLA molecules, becoming more sensitive to targeted immune cytotoxicity (57). We saw this same HLA class I upregulation in sBCs treated with inflammatory cytokines but not in the BKO variant. Others have demonstrated that ablation of the B2M gene and deletion of HLA Class I molecules of hPSCs make them susceptible to natural killer (NK) cells cytotoxicity (58–60). To circumvent this obstacle some have explored retention of the non-classical HLA Class Ib molecules, HLA-E and HLA-G, in combination with deletion of HLA Class Ia molecules (53, 60, 61). Additionally, co-expression of HLA Class Ib molecules with CD47 and/or PD-L1 have shown to further improve hPSC survival in humanized mice models (62–64). Nevertheless, transplantation of cells that are HLA Class I deficient have slower lysis kinetics relative to unmodified cells during T-cell directed immunity (54, 56) or allogenic rejection (56, 62).

One limitation of our study is the inability to fully distinguish autoimmune from allo- and xeno- rejection. However, the timeframe of the sBC survival in our model suggests that the HLA-A2 matching reduced or eliminated immediate xeno-rejection. Many sBCs cells survived for at least two weeks in our model. Others have shown that xenotransplantation of stem cell derived human beta cells into immune competent mice are fully rejected (no surviving beta cells) within one week (23). Allotransplantation of murine pancreatic islets into non-chemically induced diabetic mice with mis-matched MHC Class I also reject by 4-10 days (56, 65–69). In overtly diabetic NOD mice, syngeneic NOD islet grafts in the kidney capsule take longer to reject, approximately 8-12 days (54, 70–72). In our study, sBC clusters did not reject until days 7-14 and many viable beta cells still remained at the end of the study. The HLA Class I was matched and pre-existent islet reactive T cells were present, as demonstrated by immunohistochemistry showing substantial insulitis in the endogenous mouse pancreas. Thus, the timing of rejection in our study is more in line with other studies of autoimmunity against islet grafts than the studies of allo- or xeno-islet graft rejection.

A second limitation of our study is that, because the sBC grafts were faced with strong immune challenge immediately upon transplantation, they did not have time to engraft and mature, a process that can take several weeks. Reliable engraftment of sBCs that corrects and maintains glycemia in the face of immune challenge is critical for translation of islet replacement tolerance strategies. We did not use any systemic immune suppression such as rapamycin in our studies, which is an immunosuppressive drug that inhibits cytokine production and arrests T- cell proliferation (73, 74). This drug is used in islet allo-transplantation procedures since the development of the Edmonton protocol in 1999 (75). Short-term administration of rapamycin in allogeneic islet transplantation can protect islets from early rejection and improve later tolerance outcomes even after withdrawal of rapamycin (19–22).

In future studies, we suggest that iP and iP-BKO sBCs be first transplanted into immune deficient NSG-HLA-A2 mice and allowed sufficient time to engraft and mature, followed by adoptive transfer of T cells from diabetic NOD-cMHCI^-/–^A2. Because we have shown the T cells from NOD-cMHCI^-/–^A2 will properly traffic to grafted islets in the kidney capsule, the timing of rejection and local graft inflammation can be controlled. Such further investigation will also allow us to control the immune cell subsets participating in the rejection and test sBC and tolerance therapy dosing. In summary, our study explores critical new directions to investigate if factors such as PD-L1, HLA-I knockout, and other negative regulators of immunity can provide localized immune protection to sBC grafts in the context of islet-directed autoimmunity. Success of such a strategy for inducing local tolerance to islet grafts would further advance the availability of beta cell replacement therapy for clinical use in individuals with autoimmune diabetes without the need for systemic immune suppression.

## Data availability statement

The original contributions presented in the study are included in the article/**Supplementary Material**. Further inquiries can be directed to the corresponding author.

## Ethics statement

The animal study was reviewed and approved by University of Florida Animal Care and Use Committee.

## Author contributions

JS-G, RC-G, HR and EP conceived and designed the study, analyzed and interpreted the results and wrote the manuscript. HR and RC-G generated the iP and iP-BKO sBC. JS-G, MB and EP carried out the animal surgery and post-operative care. CR conducted diabetic animal husbandry and oversaw breeding of transgenic mice. JS-G performed the bioluminescence imaging and histological analysis. JS-G and EP performed image quantification and analysis. All authors discussed the results and commented on the manuscript. All authors contributed to the article and approved the submitted version.

## Funding

This work was supported by the Juvenile Diabetes Research Foundation (JDRF) Agreement Award 2-SRA-2019-781-S-B (EP, HR) and NIH grant R01DK132387 (EP, HR).Work in the laboratory of H.A.R. is supported by the Children`s Diabetes Foundation, NIH grants R01DK120444, a new investigator award by NIDDK-supported Human Islet Research Network (HIRN, RRID : SCR_014393; https://hirnetwork.org; UC24 DK104162), the Culshaw Junior Investigator Award in Diabetes, a CU Grubstake award and the JDRF. This work was partially support by NIH/NIDDK grant P30-DK116073 for the University of Colorado Diabetes Research Center.

## Acknowledgments

The authors would like to acknowledge the University of Florida Molecular Pathology Core, RRID : SCR_016601 for processing the frozen tissue blocks and Dr. Dave Serreze at The Jackson Lab for providing breeding pairs of NOD-cMHCI^-/–^A2 mice to initiate the study.

## Conflict of interest

HR is or has been a consultant and islets SAB member to Sigilon therapeutics, a SAB member at Prellis Biologics and consultant to Eli Lilly and Minutia.

The remaining authors declare that the research was conducted in the absence of any commercial or financial relationships that could be construed as a potential conflict of interest.

## Publisher’s note

All claims expressed in this article are solely those of the authors and do not necessarily represent those of their affiliated organizations, or those of the publisher, the editors and the reviewers. Any product that may be evaluated in this article, or claim that may be made by its manufacturer, is not guaranteed or endorsed by the publisher.
