# Supplementary material for: Human stem cell derived beta-like cells engineered to present PD-L1 improve transplant survival in NOD mice carrying human HLA class I
Source: Front Endocrinol (Lausanne). 2022 Nov 25;13:989815. doi: 10.3389/fendo.2022.989815 (PMC9732725; doi:10.3389/fendo.2022.989815)
Supplement: Supplementary file 1 [file Image_1.pdf]

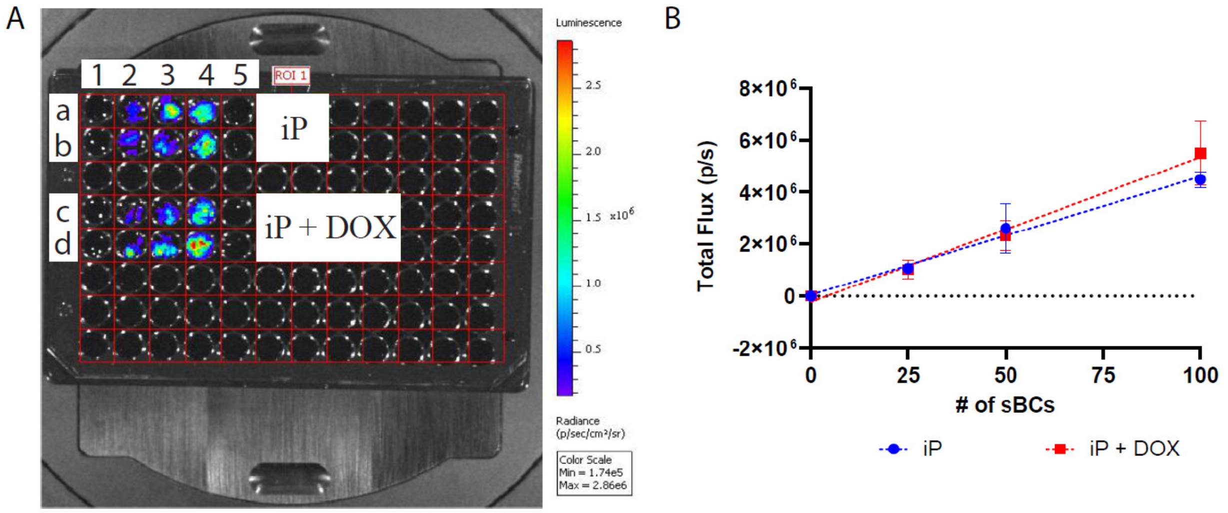

Supplementary Figure 1. Evaluation of sBC cluster bioluminescence *in vitro*. (A) iP and iP + DOX sBCs with constitutive expression of luciferase generate bioluminescence in response to D-luciferin (150 µg/mL) supplementation. The bioluminescence emitted correlated to the number of sBC clusters 1) 0 sBCs, 2) 25 sBCs, 3) 50 sBCs and 4) 100 sBCs or no bioluminescence 5) 50 sBCs with no D-luciferin. (B) Detected total flux by the IVIS® Spectrum.

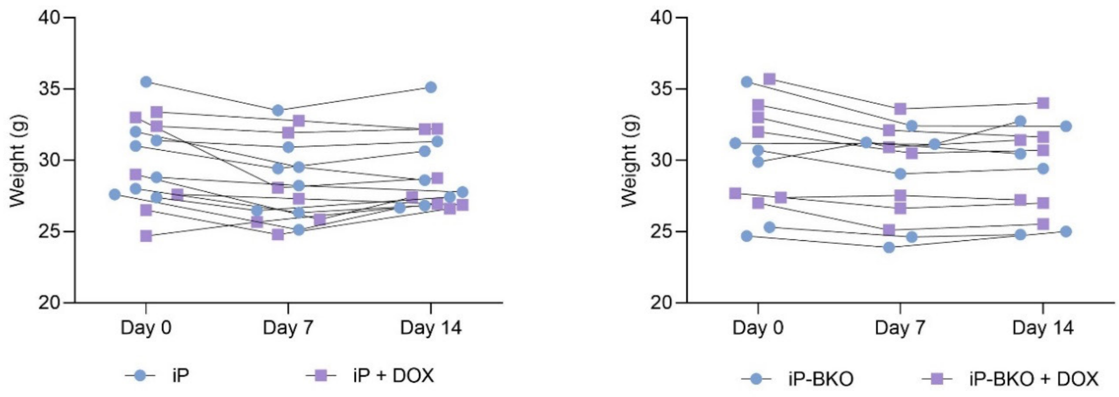

Supplementary Figure 2. Record of mouse weights for a period of two weeks after sBC transplant. Mouse weight was recorded the day of the surgery and follow-up every seven days.

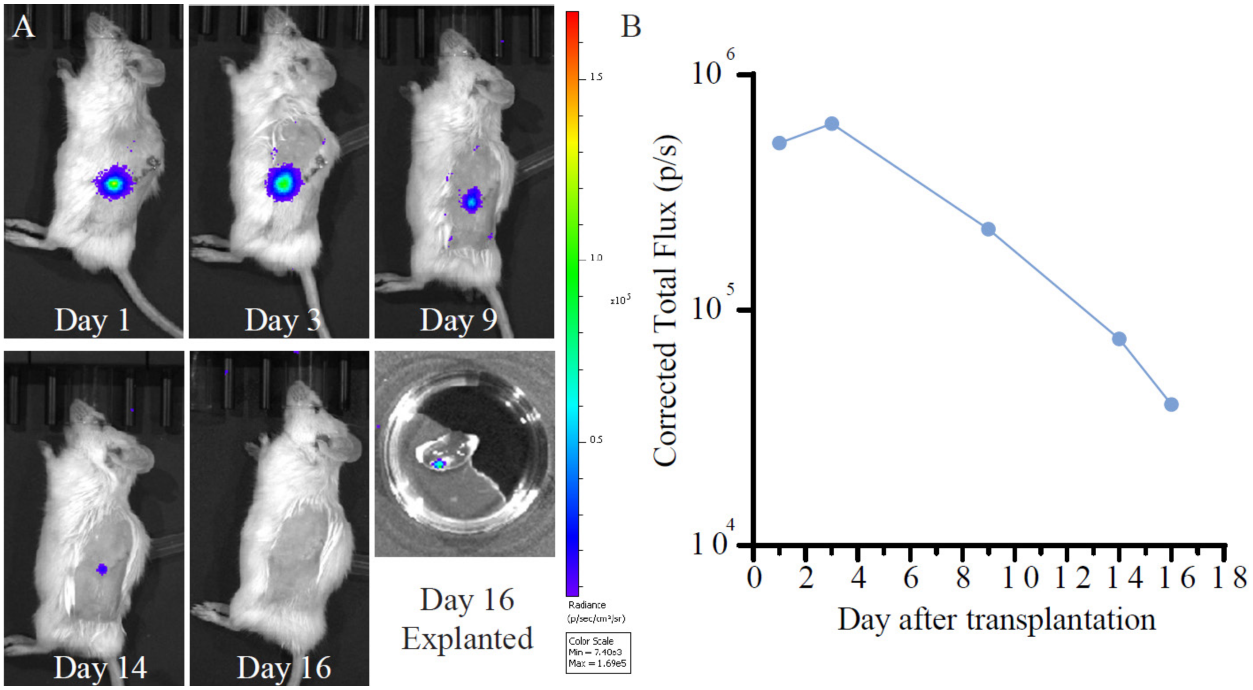

Supplementary Figure 3. One mouse of the iP-BKO sBC + DOX group had detectable bioluminescence signal for a period of two weeks. (A) Detection of bioluminescence signal *in vivo* at day 1, 3, 9, 14 and 16. In addition, explanted graft-bearing kidney had detectable bioluminescence *ex vivo* at day 16. (B) Quantification of the total flux *in vivo* up to 16 days after transplantation.

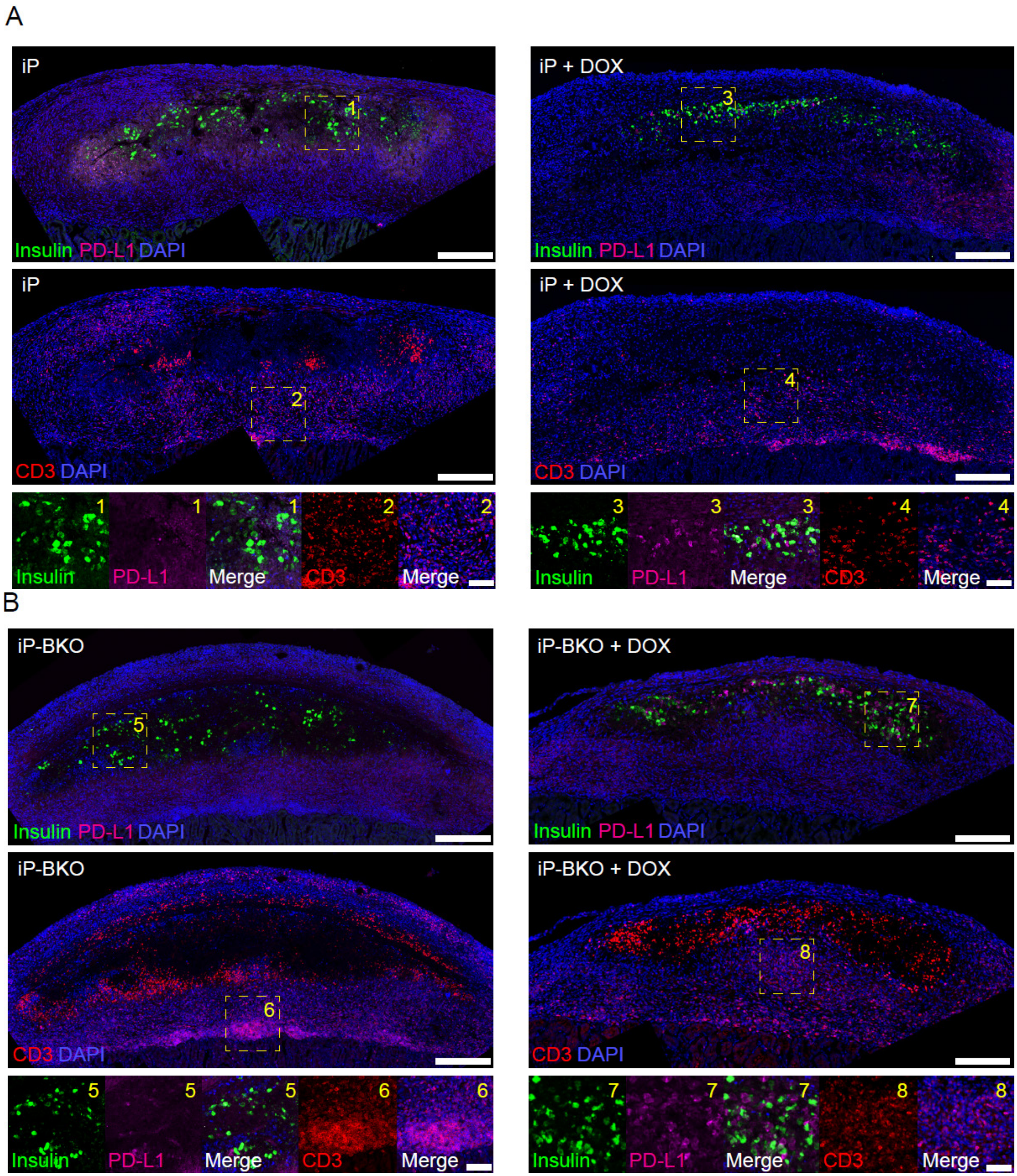

Supplementary Figure 4: Survival and maintained PD-L1 expression by sBCs two weeks after transplantation. (A) Representative tile scan MIPs of iP or iP + DOX sBCs graft-bearing kidneys immunostained for insulin, PD-L1 and CD3. (B) Representative tile scan MIPs of iP-BKO or iP-BKO + DOX sBCs graft-bearing kidneys immunostained for insulin, PD-L1 and CD3. The immunostaining for insulin and PD-L1 belong to the same sample as the CD3 immunostaining but shown separate for clarity. Scale bars size are 200 µm and 50 µm, respectively.

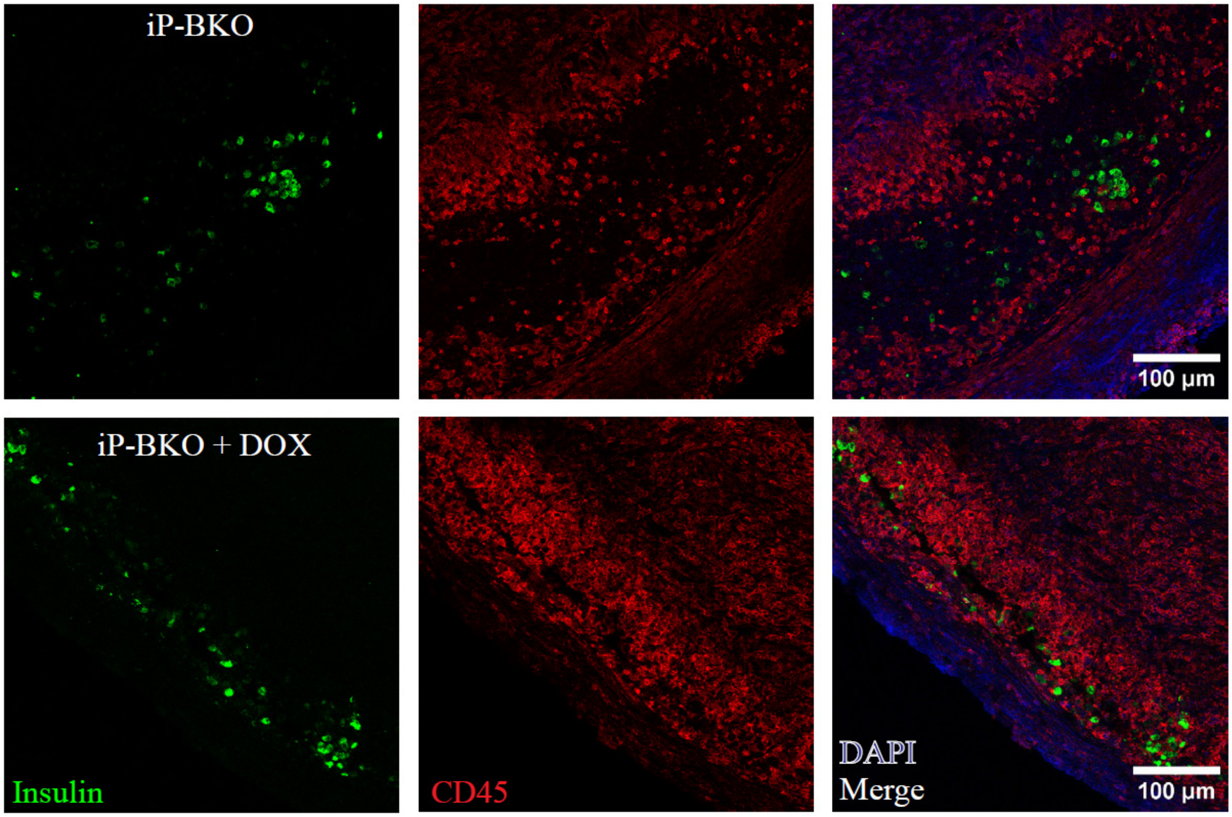

Supplementary Figure 5. Representative images of the iP-BKO sBC graft immunostained for insulin and CD45.

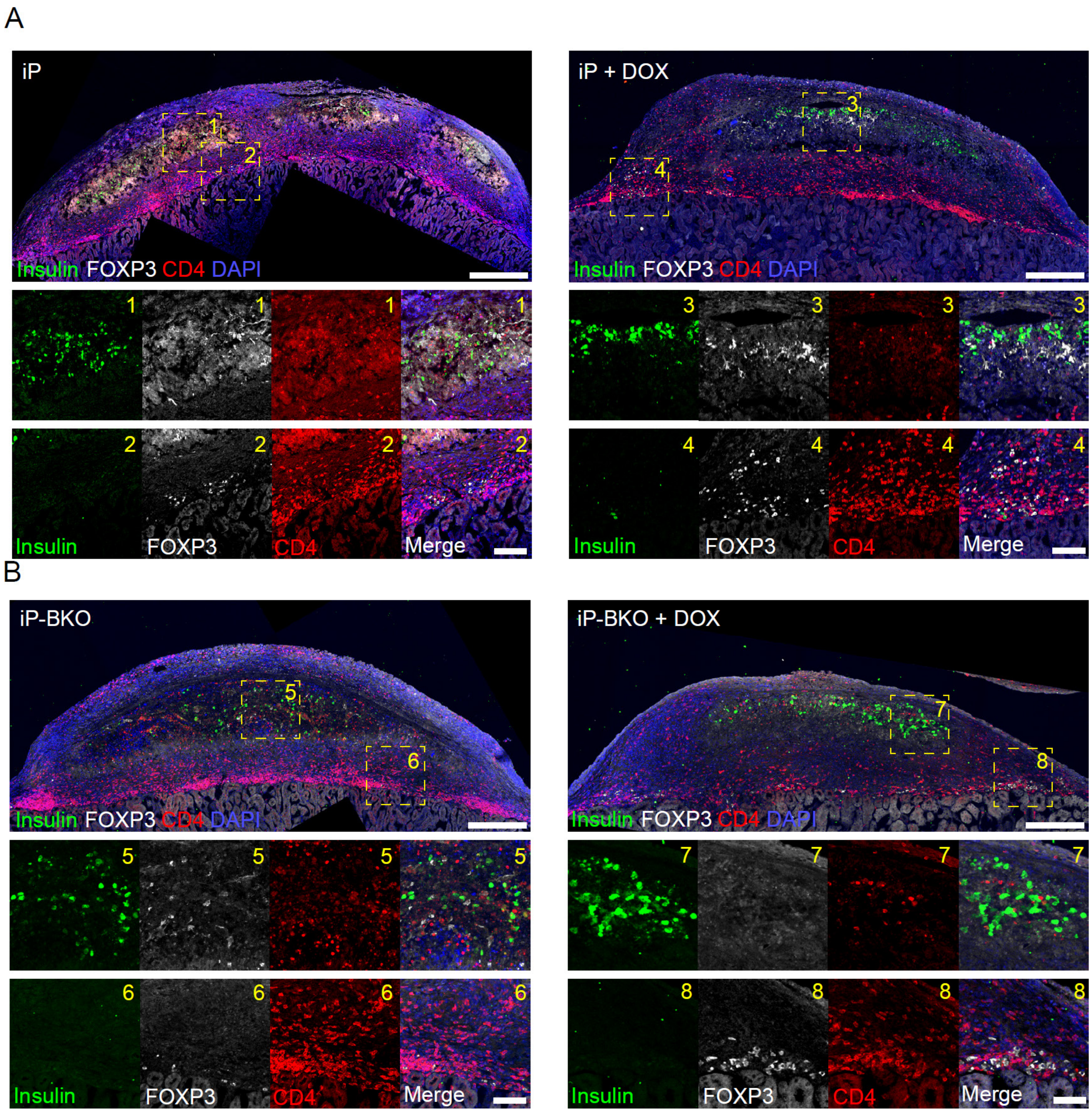

Supplementary Figure 6: FoxP3$^+$/CD4$^+$ T cells are prevalent in the sBCs graft site two weeks after transplantation. (A) Representative tile scan of iP or iP + DOX sBCs graft-bearing kidneys immunostained for insulin, FoxP3 and CD4. (B) Representative tile scan MIPs of iP-BKO or iP-BKO + DOX sBCs graft-bearing kidneys immunostained for insulin, FoxP3 and CD4. Scale bars 200 µm and 50 µm, respectively.

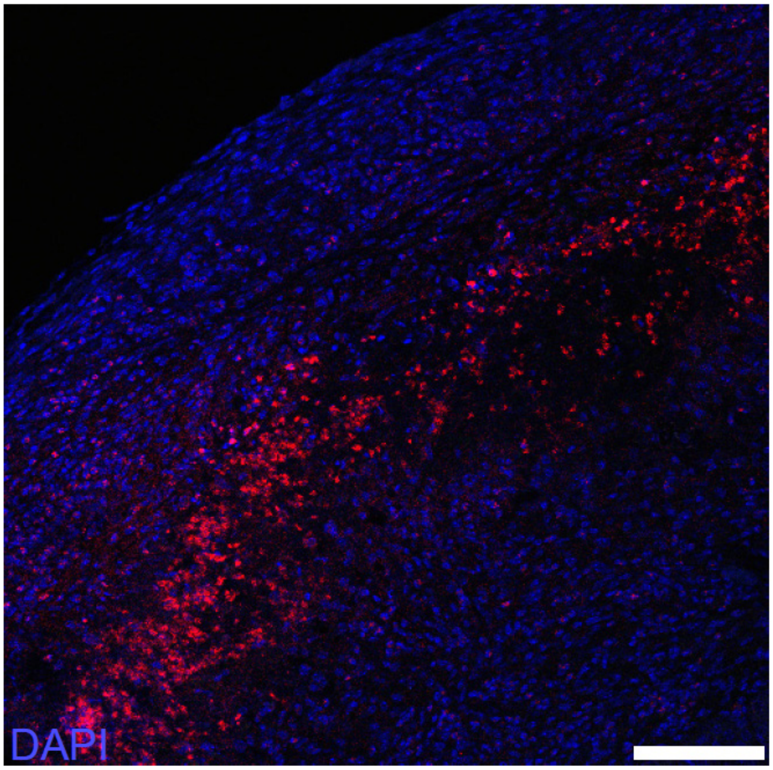

Supplementary Figure 7: Representative image of unstained graft-bearing kidney tissue. Despite no primary antibodies positive signal was observed in the red light spectrum 600-700 nm. Scale bar 100 μm

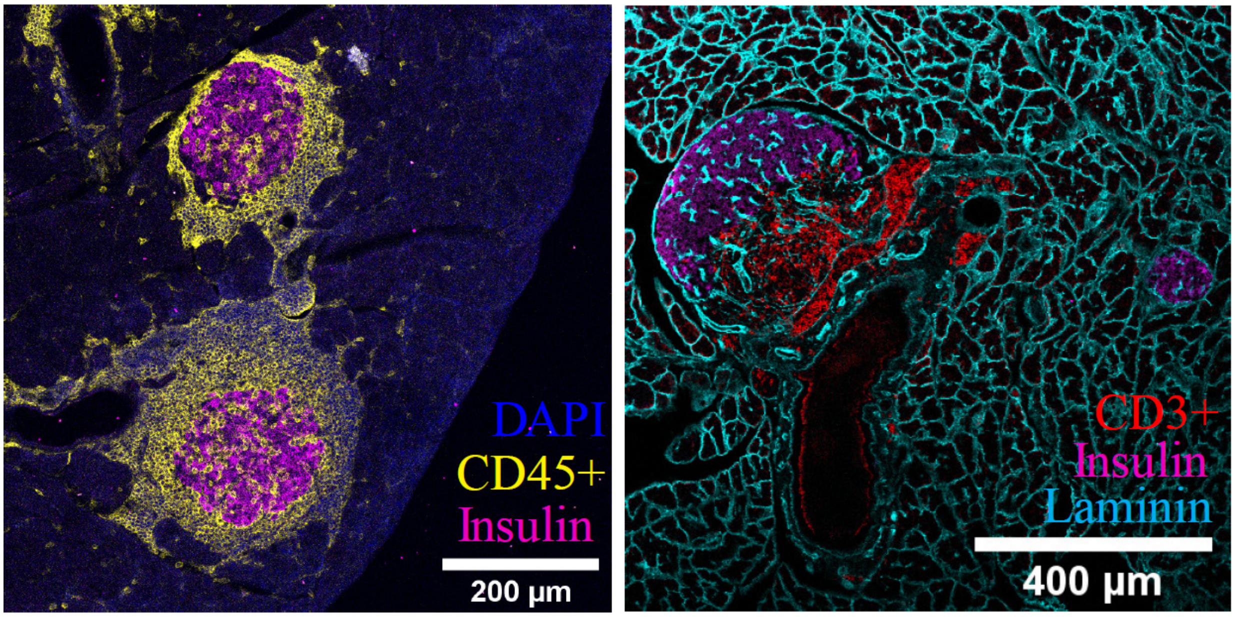

Supplementary Figure 8. Representative images of the progression of autoimmune diabetes in NOD-cMHCI$^{-/-}$-A2 mice pancreas. Infiltration of the pancreatic islets by lymphocytes (CD45^{+}) and self-reactive T-cells (CD3^{+}).

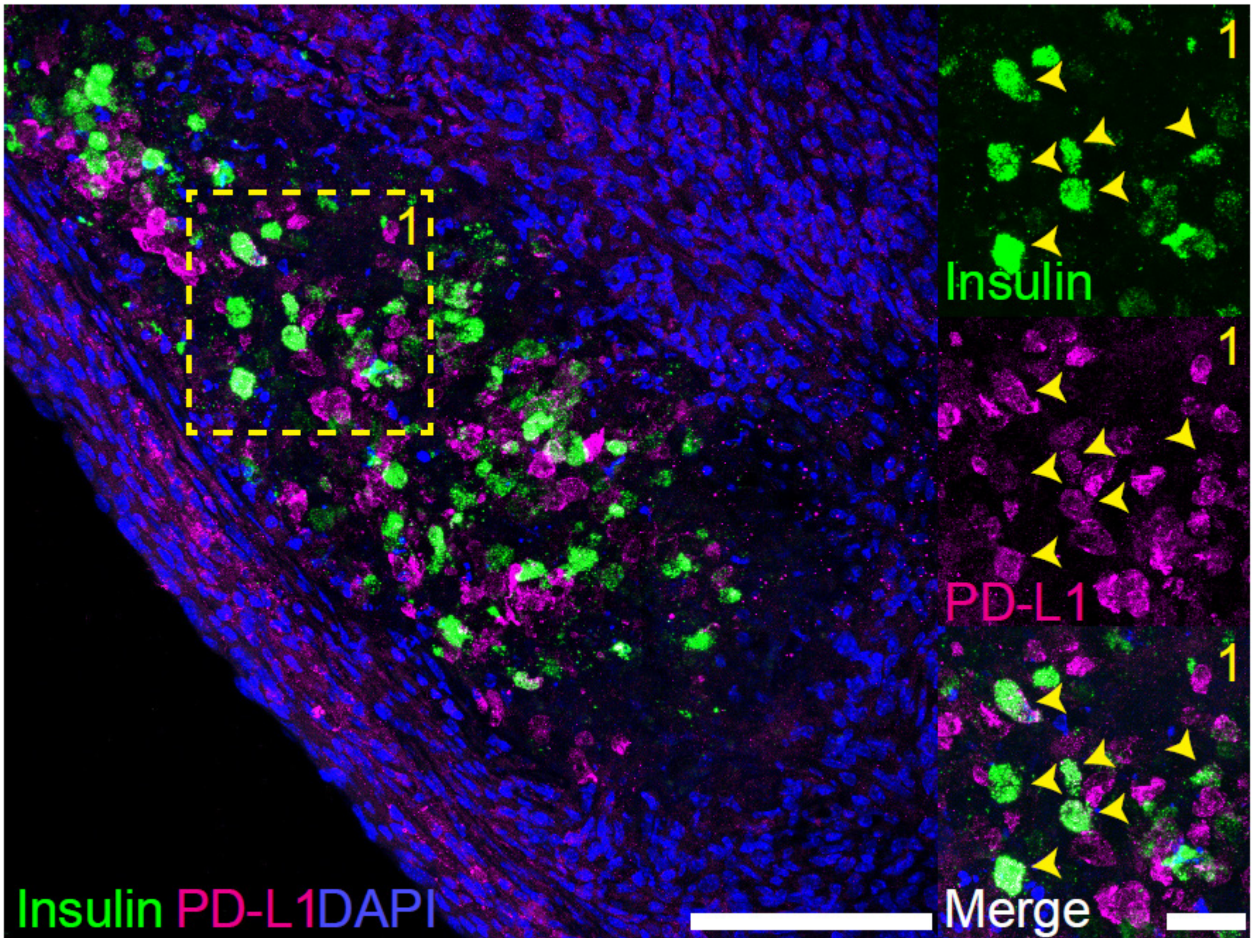

Supplementary Figure 9: Representative image of the iP-BKO sBC graft co-immunostained for insulin and PD-L1. Image was taken with a 40x oil objective. Cells positive for both insulin and PD-L1 are demonstrated with a yellow arrowhead in the insets to the right. Scale bars 100 µm and 25 µm, respectively.
